# The impact of child psychiatric conditions on future educational outcomes among a community cohort in Brazil

**DOI:** 10.1017/S2045796021000561

**Published:** 2021-10-28

**Authors:** Mauricio Scopel Hoffmann, David McDaid, Giovanni Abrahão Salum, Wagner Silva-Ribeiro, Carolina Ziebold, Derek King, Ary Gadelha, Eurípedes Constantino Miguel, Jair de Jesus Mari, Luis Augusto Rohde, Pedro Mario Pan, Rodrigo Affonseca Bressan, Ramin Mojtabai, Sara Evans-Lacko

**Affiliations:** 1 Care Policy and Evaluation Centre, London School of Economics and Political Science, Houghton Street, London WC2A 2AE, UK; 2 Universidade Federal de Santa Maria (UFSM), Avenida Roraima 1000, Building 26, Office 1446, Santa Maria 97105-900, Brazil; 3 Universidade Federal do Rio Grande do Sul (UFRGS), Rua Ramiro Barcelos 2350, Porto Alegre 90035-003, Brazil; 4 National Institute of Developmental Psychiatry for Children and Adolescents (INCT-CNPq), São Paulo, Brazil; 5 Escola Paulista de Medicina, Universidade Federal de São Paulo (UNIFESP), R. Maj. Maragliano, 241 – Vila Mariana, São Paulo SP 04017-030, Brazil; 6 Universidade de São Paulo (USP), Rua Dr Ovídio Pires de Campos 785, São Paulo 01060-970, Brazil; 7 ADHD Outpatient Program & Developmental Psychiatry Program, Hospital de Clinicas de Porto Alegre, Federal University of Rio Grande do Sul, Porto Alegre, Brazil; 8 Department of Mental Health, Johns Hopkins Bloomberg School of Public Health, 624 North Broadway, Room 797, Baltimore, MD 21205, USA

**Keywords:** Age-grade distortion, attributable risk fraction, educational attainment, grade repetition, mental health, school dropout

## Abstract

**Aims:**

Mental health problems early in life can negatively impact educational attainment, which in turn have negative long-term effects on health, social and economic opportunities. Our aims were to: (i) estimate the impacts of different types of psychiatric conditions on educational outcomes and (ii) to estimate the proportion of adverse educational outcomes which can be attributed to psychiatric conditions.

**Methods:**

Participants (*N* = 2511) were from a school-based community cohort of Brazilian children and adolescents aged 6–14 years enriched for high family risk of psychiatric conditions. We examined the impact of fear- (panic, separation and social anxiety disorder, specific phobia, agoraphobia and anxiety conditions not otherwise specified), distress- (generalised anxiety disorder, major depressive disorder and depressive disorder not otherwise specified, bipolar, obsessive-compulsive, tic, eating and post-traumatic stress disorder) and externalising-related conditions (attention deficit and hyperactivity disorder, conduct and oppositional-defiant conditions) on grade repetition, dropout, age-grade distortion, literacy performance and bullying perpetration, 3 years later. Psychiatric conditions were ascertained by psychiatrists, using the Development and Well-Being Behaviour Assessment. Propensity score and inverse probability weighting were used to adjust for potential confounders, including comorbidity, and sample attrition. We calculated the population attributable risk percentages to estimate the proportion of adverse educational outcomes in the population which could be attributed to psychiatric conditions. Analyses were conducted separately for males and females.

**Results:**

Fear and distress conditions in males were associated with school dropout (odds ratio (OR) = 2.76; 95% confidence interval (CI) = 1.06, 7.22; *p* < 0.05) and grade repetition (OR = 2.76; 95% CI = 1.32, 5.78; *p* < 0.01), respectively. Externalising conditions were associated with grade repetition in males (OR = 1.66; 95% CI = 1.05, 2.64; *p* < 0.05) and females (OR = 2.03; 95% CI = 1.15, 3.58; *p* < 0.05), as well as age-grade distortion in males (OR = 1.66; 95% CI = 1.05, 2.62; *p* < 0.05) and females (OR = 2.88; 95% CI = 1.61, 5.14; *p* < 0.001). Externalising conditions were also associated with lower literacy levels (*β* = −0.23; 95% CI = −0.34, −0.12; *p* < 0.001) and bullying perpetration (OR = 3.12; 95% CI = 1.50, 6.51; *p* < 0.001) in females. If all externalising conditions were prevented or treated, we estimate that 5.0 and 4.8% of grade repetition would not have occurred in females and males, respectively, as well as 10.2 (females) and 5.3% (males) of age-grade distortion cases and 11.4% of female bullying perpetration.

**Conclusions:**

The study provides evidence of the negative impact of psychiatric conditions on educational outcomes in a large Brazilian cohort. Externalising conditions had the broadest and most robust negative impacts on education and these were particularly harmful to females which are likely to limit future socio-economic opportunities.

## Introduction

Most psychiatric conditions begin early in life, with lifelong consequences. The global prevalence of these conditions in young people is around 13.4% (Polanczyk *et al*., 2015) and recent studies suggest this is increasing (Collishaw, 2015; Gage and Patalay, 2021). Psychiatric conditions can adversely impact future social and occupational opportunities (McDaid *et al*., 2019; Thompson *et al*., 2021). One mechanism through which psychiatric conditions may influence these long-term outcomes is by negatively impacting educational outcomes (Woessmann, 2016). Educational attainment can increase employment opportunities and formal education provides a structured setting for acquiring cognitive and socioemotional skills which are important throughout life (Heckman *et al*., 2006). A challenge for education professionals is to support young people to complete schooling at the appropriate age, without dropouts or grade repetition, and to help them develop reading and writing literacy in addition to socio-emotional skills. All of these outcomes can increase labour market participation later in life (OECD, 2014).

Previous research supports an association between poor mental health and adverse educational attainment (Vander Stoep *et al*., 2003; Breslau *et al*., 2008; Lee *et al*., 2009; Mojtabai *et al*., 2015; Veldman *et al*., 2015; Dalsgaard *et al*., 2020a; Mekonnen *et al*., 2020; Wickersham *et al*., 2021). These studies found that the population attributable risk percentage (PARP) for early high school termination due to any psychiatric condition was 10.2% in the USA (Breslau *et al*., 2008; Mojtabai *et al*., 2015), whereas lower figures based on cross-sectional data were found in low- and middle-income countries (LAMICs) such as Colombia (1.3%) and Mexico (0.5%) (Lee *et al*., 2009). Gaps exist, however, in understanding the relative impact of different types of psychiatric conditions on a range of adverse educational outcomes (Breslau *et al*., 2008; Lee *et al*., 2009, Mojtabai *et al*., 2015; Dalsgaard *et al*., 2020a).

First, most past research has focused on educational attainment (Breslau *et al*., 2008; Lee *et al*., 2009; Mojtabai *et al*., 2015). For example, Mojtabai *et al*. (2015) only examined non-completion of secondary school and university, or truncation of secondary education. Similarly, Dalsgaard *et al*. (2020a) only assessed the proportion of students taking a final examination and respective examination grades. Other indicators such as repeating a school year (or grade repetition), age-grade distortion and deficits in literacy and socio-emotional skills (which can be learned in school) may also have long-term psychosocial and occupational consequences (Jacob and Lefgren, 2009). Moreover, attainment is affected by previous grade repetition (Ikeda and Garcia, 2013) leading to age-grade distortions (i.e. being over-age for the enrolled grade); and these outcomes can also increase the risk for school dropouts (Roderick, 1994). Furthermore, literacy performance and social skills impact educational attainment (Zuilkowski *et al*., 2016) and are an educational output in their own right.

Second, as psychiatric conditions are diverse and comprise distinct phenomena, their pathways to educational attainment may also vary. However, few studies have examined the full range of psychiatric conditions (Dalsgaard *et al*., 2020a). For example, a longitudinal study in the USA by Mojtabai *et al*. (2015) did not include attention deficit and hyperactivity disorder (ADHD) – one of the most common psychiatric conditions in young people. Furthermore, past studies have not robustly adjusted for confounders, such as general intelligence, background socioeconomic status or race and ethnicity (Sirin, 2005; Deary *et al*., 2007). Finally, few studies considered differences in educational pathways according to sex (Dalsgaard *et al*., 2020a). This could be important given that males tend to have lower educational attainment and achievement rates (Voyer and Voyer, 2014; Lavoie *et al*., 2019) and higher rates of externalising conditions compared to females (Stoet and Geary, 2015), whereas females have higher rates of distress-related conditions (such as major depression) and are more likely to be unemployed in early adulthood (Zahn-Waxler *et al*., 2008; OECD, 2014) compared to males. Furthermore, Dalsgaard *et al*. (2020a) found that males with fear-, distress- or externalising-related conditions were less likely to take the final 9th grade examination than females with those conditions. However, females with fear- or externalising-related conditions had lower mean grades on the examination compared to males with those conditions.

This study addresses past limitations by first examining the impact of different psychiatric condition categories (i.e. fear-, distress- and externalising-related conditions) on a range of educational indicators beyond attainment (grade repetition, dropout, age-grade distortion, literacy performance and bullying perpetration behaviour) in young males and females separately, while implementing robust adjustment for confounders (McCaffrey *et al*., 2004). Second, we estimate the proportion of adverse educational outcomes which can be attributed to psychiatric conditions and hence, might have been avoided if these conditions were fully prevented or treated, and translated these to national figures. We hypothesised that fear-, distress- and externalising-related conditions would negatively impact subsequent educational outcomes in comparison with subjects without a psychiatric condition. Considering available evidence, we hypothesised that males would experience greater negative educational impacts due to psychiatric conditions compared to females.

## Methods

### Sample

We analysed baseline (2010–2011) and 3-year follow-up (2013–2014) data from the Brazilian High-Risk Cohort Study for Psychiatric Conditions (BHRCS), a large school-based community cohort enriched for high family risk for psychiatric conditions (Salum *et al*., 2015). The study was based on a screening stage and an assessment stage. At screening, on compulsory school registration days in 2010, all parents at state-funded schools, 22 schools in Porto Alegre and 35 in São Paulo, were invited to participate. In total, 8012 caregivers (87.3% mothers) agreed to be screened, with a modified version of the Family History Screen (FHS) by lay interviewers (Weissman *et al*., 2000). The FHS is a structured interview used to screen all family members for psychiatric conditions based on DSM-IV. We generated a family liability index that expresses the percentage of family members who screened positive for a psychiatric condition, adjusted for degree of relatedness (mother, father and sibling counts as 1.0, half sibling is 0.5). For full family liability index description, see Salum *et al*. (2015). We recruited two subgroups (one child per family): a high-risk sub-sample based on the family liability index (*n* = 1553) and a random subsample based on all eligible children (*n* = 958). These families (*n* = 2511) were selected for full household assessment by lay interviewers (parent interview) and trained psychologists (child interview) at baseline (6–14 years) and at follow-up (9–17 years, 80% retention). Participation was associated with higher maternal education, socioeconomic group (SEG), living in Porto Alegre, and anxiety-related conditions at baseline (Pan *et al*., 2017).

This study was approved by the National Research Ethics Commission (Comissão Nacional de Ética em Pesquisa), University of São Paulo and Federal University of Rio Grande do Sul ethics committees. Written informed consent was obtained from parents and participants that were able to read, write and clearly understand the written consent.

### Psychiatric assessment

Current psychiatric conditions were assessed at baseline using parent-report on the Brazilian–Portuguese version of the Development and Well-being Assessment (DAWBA) (Goodman *et al*., 2000; Fleitlich-Bilyk and Goodman, 2004). The DAWBA is a semi-structured interview used to generate current DSM-IV diagnoses. Diagnostic probabilities were generated based on responses to lay interviewers. Verbatim responses as well as structured answers were then evaluated by nine trained psychiatrists who confirmed, refuted or altered initial computerised diagnostic probabilities to determine final diagnosis (overall agreement = 91%). Inconclusive cases were discussed with researchers and senior psychiatrists as described previously (Salum *et al*., 2015).

Based on previous literature (Mojtabai *et al*., 2015; Martel *et al*., 2017), conditions were grouped into three broad categories: fear-related conditions (including panic, separation and social anxiety disorder, specific phobia, agoraphobia and anxiety conditions not otherwise specified), distress-related conditions (including generalised anxiety disorder, major depressive disorder and depressive disorder not otherwise specified, bipolar, obsessive-compulsive, tic, eating and post-traumatic stress disorder) and externalising conditions (including ADHD, conduct and oppositional-defiant conditions). A dummy comorbidity variable was generated for individuals with diagnoses belonging to two or more broad categories (Table 1 for details regarding diagnostic overlap). Participants with baseline autism (*n* = 15) were excluded due to divergent literature as to which broad group they might belong.

**Table 1.** Psychiatric conditions at baseline (2010–2011) by each group

Conditions	Any fear (*n* = 212)		Any distress (*n* = 135)		Any externalising (*n* = 358)		Cross-category comorbidity (*n* = 103)		Total (*n* = 587)	
	*n*	%	*n*	%	*n*	%	*n*	%	*n* *=* 587	%
Separation anxiety	72	34.0					27	37.5	72	12.3
Specific anxiety	86	40.6					24	27.9	86	14.7
Social anxiety	26	12.3					7	26.9	26	4.4
Panic	1	0.5					1	100.0	1	0.2
Agoraphobia	4	1.9					1	25.0	4	0.7
Other anxieties	44	20.8					12	27.3	44	7.5
Depression			86	63.7			42	48.8	86	14.7
Bipolar			5	3.7			4	80.0	5	0.9
PTSD			23	17.0			14	60.9	23	3.9
OCD			7	5.2			2	28.6	7	1.2
Psychosis			1	0.7			0	0.0	1	0.2
Eating disorder			10	7.4			6	60.0	10	1.7
Tic disorder			19	14.1			8	42.1	19	3.2
Any ADHD					270	75.4	54	20.0	270	46.0
Any Conduct					49	13.7	16	32.7	49	8.3
Any ODD					131	36.6	33	25.2	131	22.3

PTSD, post-traumatic stress disorder; OCD, obsessive-compulsive disorder; ADHD, attention deficit and hyperactivity disorder; ODD, oppositional-defiant disorder.

*Note*: Cross-category comorbidity group was defined as subjects with conditions belonging to more than one diagnostic group (comorbidity between categories). Percentages are calculated by *n*/total cases on condition category. Percentage in the cross-category column indicates the percentage of subjects with a cross-category comorbidity within the specific condition. Percentages may not result in 100% due to within-category comorbidity.

### Educational outcomes

We considered educational outcomes reported at follow-up which had occurred since baseline. Caregivers reported on their child's grade repetition and school dropout (including expulsion). Age-grade distortion was calculated for participants reporting grade repetition, dropout or expulsion since baseline, but still enrolled in education. For these participants, age-grade distortion was measured in years and calculated by subtracting the participant's current age from the expected age range for the current school grade. For example, if a child was 12–13 years and enrolled in 5th grade (expected age 10–11 years), we estimated an age-grade distortion of 2 years. For subjects not currently enrolled in school but reporting grade repetition or dropout since baseline, to be conservative, we attributed the value of 1 year.

Reading and writing literacy was measured via the School Performance Test (‘Teste de Desempenho Escolar’ – TDE) (Salum *et al*., 2015). We used standardised factor scores based on confirmatory factor analysis described in online Supplementary material.

We included bullying perpetration as an education-related outcome as it is an example of maladaptive peer interaction and a proxy for poor socioemotional skills (Rose *et al*., 2011; Espelage *et al*., 2018). Bullying perpetration was assessed at follow-up in the interview with a trained psychologist using one question after the following prompt: ‘We say that a person is bullied when another person or group of people say or do unpleasant and mean things to him/her. It's also bullying when a person is repeatedly teased in a way they don't like. Examples of bullying are giving mean nicknames, humiliating, assaulting or hurting a helpless classmate, pushing, breaking and stealing belongings, chasing, isolating, ignoring, causing suffering, etc.’. After that, we asked: ‘Have you bullied another child in the past 12 months?’. Responses were ‘Yes’, ‘No’, ‘I don't know’.

### Covariates

We adjusted the analyses for the following potentially confounding baseline variables on the association between psychiatric condition and educational outcomes: age; SEG; race/ethnicity, categorised as white and non-white (black, mixed, Asian and indigenous participants); intelligence (IQ); study site (due to differences in state-level legislation on retention and prevalence of psychiatric conditions) and the dummy comorbidity variable described above.

SEG was assessed using the classification from the ‘Associação Brasileira de Empresas de Pesquisa’ (Brazilian Association of Research Companies) (ABEP, 2010). Classification is made through a composite score comprising the main caregiver's educational level and household assets and conditions. A/B represent the high/comfortable class; C is considered middle class and D/E the lowest social class. SEG was considered as lower (groups C, D and E) compared against upper class (A/B) in the propensity score weighting calculation and adjusted regression analysis.

IQ was assessed by trained psychologists with the vocabulary and block design subtests of the Wechsler Intelligence Scale for Children, 3rd edition – WISC-III (Wechsler, 2002), using the Tellegen and Briggs method (Tellegen and Briggs, 1967). We applied Brazilian norms (Nascimento and Figueiredo, 2002) and standardised the scores by age and gender.

### Statistical analysis

We estimated the impact of baseline psychiatric conditions on educational outcomes at 3-year follow-up using unadjusted and adjusted regression models and using schools as the primary sampling units. Maternal education, any anxiety condition and study site predicted response at follow-up in the present sample (Pan *et al*., 2017). Therefore, we used these variables to compute inverse probability weights (IPWs) to address sample attrition in all regression analyses. Adjusted analyses included covariates (described above), baseline assessment of the outcome being estimated, IPWs and also propensity score weights (PSWs), to balance participants with and without psychiatric conditions for baseline characteristics (McCaffrey *et al*., 2004), conducting, therefore, a doubly robust approach for the regression models (Kang and Schafer, 2007; Elze *et al*., 2017). All regression models were estimated using the *survey* command in Stata 15.1 (StataCorp, College Station, TX).

PSWs were derived using generalised boosted modelling in the R *twang* package (Ridgeway *et al*., 2017). This generates estimates of exposure probability based on covariates and fits several models using a regression tree and then merges predictions computed by each model. It also balances groups for covariate missingness. Interaction trees were set to 5000, shrinkage to 0.01 and two-way depth of interaction was used to minimise prediction errors by means of subsampling strategies (McCaffrey *et al*., 2004). Balance in covariates between groups (e.g. belonging to a psychiatric condition category or not) was ascertained comparing standardised mean differences. PSWs were calculated separately for gender and psychiatric condition groups. Potential baseline confounders included in PSW estimation are described above; these were the same covariates used in the regression models. PSWs and IPWs were multiplied to generate final weights used in all adjusted models.

### Population attributable risk percentage

We calculated PARPs for each educational outcome (grade repetition, school dropout, age-grade distortion and bullying perpetration) due to psychiatric condition category. Estimated PARPs can be interpreted as a percentage of adverse educational outcomes in the total population attributable to psychiatric conditions (and the percentage potentially preventable by successfully treating or preventing baseline psychiatric conditions). The PARP was computed using *punaf* command in Stata 15.0 (StataCorp, College Station, TX), based on weighted predicted probabilities from regression models. ‘Punaf’ generates estimates based on two scenarios: first, using predicted probabilities from the model and second, if all participants did not have a psychiatric condition. The ratio of predicted prevalence estimates from the two scenarios was then used to calculate PARPs. As we aimed to generalise to the Brazilian population, we included an additional sampling weight for PARP calculations. This sampling weight fully accounted for the family liability oversampling (see sample description). The methods and results can be found in the Supplemental material in Martel *et al*. (2017). This allowed us to reweight our sample back to general population figures, achieved by setting a survey design in Stata.

For educational outcomes, we used data from the National Institute of Educational Studies and Research database to estimate the number of Brazilians to which PARPs would translate. Details of estimates are described in online Supplementary material.

## Results

### Participant characteristics

Table 2 describes sample characteristics, by gender, at baseline and follow-up. Males had a higher proportion of externalising-related conditions and grade repetition, age-grade distortion, bullying perpetration and lower literacy at baseline and follow-up. Table 1 describes the distribution of mental health condition categories at baseline. Fear-related conditions mostly comprised of specific phobia (40.6%), distress-related conditions major depression (63.7%) and externalising-related conditions ADHD (75.4%). Individuals with distress-related conditions had the highest cross-category comorbidity (50.3%).

**Table 2.** Sample description, by gender at baseline (2010–2011) and follow-up (2013–2014)

Baseline	Males	Females	*p*-value
	*n* = 1375	*n* = 1136	
Any fear-related condition	114 (8.3)	98 (8.6%)	0.819
Any distress-related condition	67 (4.9%)	68 (6.0%)	0.253
Any externalising condition	228 (16.6%)	130 (11.4%)	<0.001
Comorbidity of condition categories	61 (4.4%)	42 (3.7%)	0.407
Age in years (s.d.)	10.1 (1.88)	10.3 (1.93)	0.135
Site			
Porto Alegre	662 (48.1%)	593 (52.2%)	0.047
São Paulo	713 (51.9%)	543 (47.8%)	
Race/ethnicity			
White	811 (59.0%)	708 (62.3%)	0.412
Black	154 (11.2%)	110 (9.7%)	
Mixed (black and white)	397 (28.9%)	309 (27.2%)	
Indigenous	5 (0.4%)	6 (0.5%)	
Asian	4 (0.3%)	1 (0.1%)	
SEG			
Lower class (D/E)	37 (2.69%)	41 (3.61%)	0.048
Middle class (C)	764 (55.6%)	671 (59.1%)	
Upper class (A/B)	574 (41.7%)	424 (37.3%)	
IQ in *z*-scores (s.d.)	0.07 (1.02)	−0.08 (0.97)	<0.001
Grade repetition	300 (21.9%)	196 (17.3%)	0.005
School dropout	31 (2.3%)	24 (2.1%)	0.916
Age-grade distortion	268 (19.5%)	170 (15.0%)	0.003
Literacy factor score (s.d.)	−0.56 (0.91)	−0.43 (0.89)	0.001
Bullying perpetration	192 (15.8%)	110 (10.9%)	<0.001
Follow-up	Males	Females	
	*n* = 1125	*n* = 885	
Grade repetition	344 (30.6%)	223 (25.2%)	0.009
School dropout	67 (6.0%)	46 (5.2%)	0.526
Age-grade distortion	313 (27.8%)	205 (23.2%)	0.021
Age-grade distortion (years)			
1	37 (3.3%)	30 (3.4%)	
2	83 (7.4%)	34 (3.8%)	
3	83 (7.4%)	65 (7.3%)	
4	56 (5.0%)	46 (1.9%)	
5	29 (2.6%)	17 (1.9%)	
6	25 (2.2%)	13 (1.5%)	
Literacy factor score (s.d.)	0.01 (0.62)	0.13 (0.64)	<0.001
Bullying perpetration (self-report)	143 (13.6%)	60 (7.3%)	<0.001

SEG, socioeconomic group measured using ABEP score; IQ, Intelligence quotient.

Potential confounders were adequately balanced (standardised mean difference <0.2) between weighted groups following application of PSWs (see online Supplementary material, Tables S1a to S3b).

### Relationship between psychiatric conditions and adverse educational outcomes

Table 3 presents PSW-adjusted estimates, stratified by gender. Unadjusted results estimates are presented in online Supplementary Table S4. Full results including covariates are presented in online Supplementary Tables S5a to S7b.

**Table 3.** Adjusted association of baseline psychiatric condition category with subsequent educational outcomes by gender

Gender/psychiatric condition category	Grade repetition		School dropout		Age-grade distortion (years)		Age-grade distortion (binary)		Literacy		Bullying perpetration	
	OR	CI	OR	CI	IRR	CI	OR	CI	*β*	CI	OR	CI
Females												
Any fear	1.42	[0.82, 2.46]	0.37	[0.09, 1.43]	1.22	[0.80, 1.87]	1.33	[0.72, 2.48]	−0.06	[−0.17, 0.04]	2.23	[0.84, 5.94]
Any distress	0.96	[0.51, 1.81]	1.61	[0.48, 5.35]	0.89	[0.58, 1.36]	0.85	[0.43, 1.68]	−-0.04	[−0.17, 0.08]	2.08	[0.79, 5.47]
Any externalising	2.03*	[1.15, 3.58]	0.59	[0.16, 2.18]	2.02**	[1.28, 3.17]	2.88***	[1.61, 5.14]	−0.23***	[−0.34, −0.12]	3.12**	[1.50, 6.51]
Males												
Any fear	0.93	[0.46, 1.87]	2.76*	[1.06, 7.22]	1.01	[0.64, 1.60]	1.25	[0.72, 2.16]	−0.12	[−0.24, 0.01]	0.57	[0.22, 1.52]
Any distress	2.76**	[1.32, 5.78]	3.16	[0.79, 12.64]	1.25	[0.77, 2.05]	1.85	[0.92, 3.71]	0.16	[−0.01, 0.32]	2.02	[0.77, 5.33]
Any externalising	1.66*	[1.05, 2.64]	1.24	[0.51, 3.01]	1.39*	[1.06, 1.83]	1.66*	[1.05, 2.62]	0.00	[−0.09, 0.09]	1.36	[0.74, 2.47]

OR, odds ratio; CI, 95% confidence interval; IRR, incidence rate ratio.

*Note*: Models were weighted using IPWs for non-response in follow-up and propensity score weighting using condition categories as treatment variable, compared without any psychiatric condition. Covariates estimates can be found in the Supplementary material and includes baseline age, site, SEG, race/ethnicity and intelligence quotient, comorbidity with another condition category and baseline level of the specific outcome.

*p < 0.05; **p < 0.01.

Among males, fear-related conditions were associated with higher odds of school dropout, and distress-related conditions were associated with higher odds of grade repetition (Table 3). Externalising-related conditions were associated with grade repetition and age-grade distortion for males and females, and with literacy and bullying perpetration in females (Table 3).

Comorbidity between fear-, distress- and externalising-related condition categories was included as a covariate in regression models and associated estimates are presented in online Supplementary Tables S6a to S8b. Females with a comorbid condition in addition to fear-related or distress-related condition had higher odds for school dropout and age-grade distortion, respectively (online Supplementary Tables S5a and S6a). Males with fear-related or distress-related conditions who met criteria for at least one other condition category had higher odds of bullying perpetration and low literacy levels respectively (online Supplementary Tables S5b and S6b).

### Population attributable risk percentage

Table 4 presents the PARP for each psychiatric condition category and estimated number of adverse educational outcomes in Brazil (reference year 2014) avoided if all conditions within categories were treated or prevented and assuming a causal relationship between psychiatric conditions and educational outcomes. When applying estimates from Table 3, weighted for high-risk sampling, we found the highest PARPs were for bullying perpetration among females with externalising diagnoses (PARP = 11.4%, 95% confidence interval (CI) = 1.5, 20.3).

**Table 4.** PARP of diagnostic categories for adverse educational outcomes

	Grade repetition		School dropout		Age-grade distortion		Bully perpetrator	
	Estimate	95% CI	Estimate	95% CI	Estimate	95% CI	Estimate	95% CI
*Fear conditions*								
Female								
PARP	1.9%	−1.2, 9	**−5**.**6%**	−9.8, −1.5	1.7%	−2.2, 5.5	4.5%	−2.6, 11.1
Population (in 1000s)	13 253		13 253		13 253		13 253	
% of outcome	11.7%		5.4%		28.7%		15.6%	
Estimated nationwide avoided cases (in 1000s)	29	−19, 76	**−40**	−70, −11	66	−84, 210	93	−54, 229
Male								
PARP	−0.4%	−4.1, 3.2	8.3%	−2.0, 17.5	1.4%	−2.2, 4.8	−3.9%	−10.2, 2.0
Population (in 1000s)	13 581		13 581		13 581		13 581	
% of outcome	11.7%		5.4%		28.7%		24.2%	
Estimated nationwide avoided cases (in 1000s)	−6	−66, 51	61	−15, 128	53	−85, 187	−129	−337, 67
*Distress conditions*								
Female								
PARP	−0.1%	−2.1, 1.8	2.9%	−5.3, 10.4	−0.5%	−2.5, 1.5	2.6%	−1.8, 6.7
Population (in 1000s)	13 253		13 253		13 253		13 253	
% of outcome	11.7%		5.4%		28.7%		15.6%	
Estimated nationwide avoided cases (in 1000s)	−2	−33, 28	20	−38, 74	−19	−95, 56	53	−37, 140
Male								
PARP	**2.6%**	0.3, 4.9	2.6%	−1.5, 6.6	1.5%	−0.5, 3.5	1.8%	−1.0, 4.4
Population (in 1000s)	13 581		13 581		13 581		13 581	
% of outcome	11.7%		5.4%		28.7%		24.2%	
Estimated nationwide avoided cases (in 1000s)	**42**	4, 78	19	−11, 49	59	−18, 135	58	−32, 146
*Externalising conditions*								
Female								
PARP	**5.0%**	0.7, 9.2	−4.3%	−15.2, 5.5	**10.2%**	3.2, 16.6	**11.4%**	1.5, 20.3
Population (in 1000s)	13 253		13 253		13 253		13 253	
% of outcome	11.7%		5.4%		28.7%		15.6%	
Estimated nationwide avoided cases (in 1000s)	**78**	12, 142	−31	−109, 39	**386**	123, 630	**236**	32, 420
Male								
PARP	**4.8%**	0.2, 9.2	2.6%	−9.5, 13.4	**5.3%**	0.1, 10.2	4.1%	−4.8, 12.2
Population (in 1000s)	13 581		13 581		13 581		13 581	
% of outcome	11.7%		5.4%		28.7%		24.2%	
Estimated nationwide avoided cases (in 1000s)	**76**	2, 146	19	−70, 98	**205**	2, 398	135	−157, 402

*Note*: Estimated avoided cases = PARP × Population in 1000s × % of outcome. PARP estimates were derived from the adjusted regression models (Table 3) and weighted for high-risk oversampling to balance for random sample. To estimate the number of people that would have benefited if mental health conditions would have been avoided, we used data from the Instituto Nacional de Estudos e Pesquisas Educacionais (INEP) to calculate the population enrolled in schools at age range for the follow-up sample, which ranged from 6 to 17 years (primary and secondary education age range) as well as the proportion of outcomes. Therefore, ‘Population’ refers to the number of males (18 661 803) and females (18 211 089) enrolled in schools multiplied by the proportion of state-funded urban students in 2014 (72.78%). The ‘% of outcomes’ were extracted from the 2014 Brazilian educational census (http://portal.inep.gov.br/web/guest/indicadores-educacionais) for grade repetition, dropout and age-grade distortion. In **bold**, *p* < 0.05.

Applying school participation estimates for 2014 in Brazil, described in online Supplementary material (p. 3), we estimate that for the Brazilian population, 154 000 cases of grade repetition (4.9% of all school repetition, 95% CI = 0.4, 9.2), 591 000 cases of age-grade distortions (7.7% of all cases, 95% CI = 1.6, 13.6) and 236 000 cases of bullying perpetration (4.4% of all cases, 95% CI = 0.6, 7.8) would have been avoided if externalising conditions were prevented or treated. As hypothesised, some differences according to gender were found, Table 4 presents estimates for males and females separately.

## Discussion

Psychiatric conditions can have multiple short- and long-term impacts on individuals and society (Mojtabai *et al*., 2015; McDaid *et al*., 2019; Thompson *et al*., 2021). Here, we demonstrate how early psychiatric conditions can broadly impact education. Our findings highlight the independent impact of psychiatric conditions, particularly externalising conditions, on grade repetition and age-grade distortion for males and females. Moreover, we found that females experienced additional impacts associated with having an externalising condition including lower literacy ability and higher bullying perpetration. On the contrary, fear- and distress-related conditions were associated with school dropout and grade repetition in males only. Our study used PSWs to balance the condition groups for confounders, likely making our estimates more conservative; yet increases confidence of direct independent impacts. Finally, to contextualise our findings from a population perspective, we estimated that if all psychiatric conditions would have been treated or prevented, 196 000 students with grade repetition, 591 000 students with age-grade distortion and 236 000 students with bullying perpetration behaviour would have been avoided in Brazil in a 3-year period, further supporting them towards reaching future life goals. This provides evidence for the importance of effective treatment and prevention of psychiatric conditions for better educational outcomes.

### Impact of externalising conditions on educational outcomes

Our findings suggest that externalising conditions had the most robust negative impacts across educational outcomes for both genders but especially for females. Individuals with externalising conditions experienced more grade repetition and age-grade distortion compared to those without these conditions. This is supported by previous literature from high-income countries showing externalising conditions are associated with a lower probability of educational completion and literacy difficulties in primary and secondary education (Carroll *et al*., 2005; Breslau *et al*., 2008; Lee *et al*., 2009; Mojtabai *et al*., 2015; Dalsgaard *et al*., 2020a). Our findings demonstrate this in one of the largest LAMICs, using longitudinal data, which were not previously reported (Lee *et al*., 2009), where welfare safety nets are rare and gainful employment resulting from educational attainment can mitigate severe poverty and exclusion (Patel *et al*., 2018).

Nonetheless, the impacts of externalising conditions on promoting lower literacy and higher bullying perpetration were found in females only. Previous research suggests externalising conditions are more likely to be under-detected in females compared to males (Dalsgaard *et al*., 2020b) thus, females with these conditions may also be perceived as less impaired and encouraged to take the exams, ultimately leading to lower performance in literacy and math (Dalsgaard *et al*., 2020a). This may not be the case in the current study because all participants were assessed for psychopathology and literacy skills. Therefore, this could be explained in at least three alternative ways. First, this may be due to a floor effect among males who already had lower literacy and were more prone to bullying compared to females at baseline. Second, externalising conditions may develop earlier in males than in females (Hill *et al*., 2006) and their literacy and socioemotional skills could be affected earlier on by these conditions (Carroll *et al*., 2005). A third possibility is that externalising behaviours such as impulsivity, aggression and oppositionality may be less socially acceptable in Brazilian females compared to males (Burity, 2020). Thus, the association between externalising conditions with lower literacy and bullying perpetration may be driven by stigma and social non-acceptance, which, if addressed, could minimise these impacts. Whatever the mechanism, female students with externalising conditions may need special support to prevent, detect and treat externalising conditions early on to avoid lower literacy and higher rates of bullying behaviour.

### Impact of fear- and distress-related conditions on educational outcomes

We also found gender differences among those with fear- and distress-related conditions whereby males (but not females) were more likely to experience school dropout and grade repetition, respectively. This further increases the gender disparity already experienced by males who have lower educational attainment than females (OECD, 2014; Voyer and Voyer, 2014). Previous evidence suggests that young males are particularly impacted by fear- and distress-related conditions. They have greater psychological impairment and lower levels of academic attainment compared to females with the same conditions (Dunn and Goodyer, 2006; Riglin *et al*., 2013; Dalsgaard *et al*., 2020a). Compared with females, fear- and distress-related conditions are less likely to be detected and treated in males and consequently, they may be less likely to receive support and understanding from teachers and parents, who may perceive their poor performance as evidence of being lazy or deliberately unruly (Lavoie *et al*., 2019; Dalsgaard *et al*., 2020a). Furthermore, it is possible that males with fear- and distress-related conditions (e.g. specific phobias, depression, etc.) could be stigmatised because in many societies, Brazil included, these symptoms would not be expected or tolerated in young males. The mediation role of stigma and social norms in psychiatric conditions and educational outcomes deserves further scrutiny.

### Population health and policy implications

To contextualise the population impact of psychiatric conditions, we calculated PARPs for each educational indicator. Previous research has estimated that up to 10.2% of early school termination could be attributed to psychiatric conditions (Vander Stoep *et al*., 2003; Breslau *et al*., 2008), including a cross-sectional study using data from seven LAMICs (0–5.4%) (Lee *et al*., 2009). Our study provided estimates linking psychiatric conditions to prior educational indicators that lead to early school termination. Moreover, after correcting for the high-risk sampling, we found that 5.6% (95% CI = 1.5, 9.8) of school dropouts were avoided in females with fear-related conditions. This might be explained by the fact that females with these conditions tend to be more frequently detected and potentially treated (Dalsgaard *et al*., 2020b).

As a practical application of our estimates, we consider the case of ADHD in Brazil. Among those with ADHD, it is estimated that only 20% receive medication treatment (Mattos *et al*., 2012). If treatment coverage increased to 30% and assuming treatment efficacy of 70% (Mattos *et al*., 2012) and considering the proportion of individuals with a diagnosis of ADHD in the externalising condition group (75.4%), according to our PARP estimates, we can estimate that 8128 (95% CI = 739, 15 253) children and adolescents would not have repeated the school year from 2013 to 2014 in state-funded urban schools (78 000 female + 76 000 male repetitions due to externalising conditions × 10 percent-point increase in ADHD coverage × 70% treatment efficacy × 75.4% of ADHD cases in the externalising group). In this example, this represents a group of young people that would have completed education on time, with their peers, with less dropout risk and a greater chance of completing higher education, ultimately leading to better employment, earnings and future life opportunities (Jacob and Lefgren, 2009; OECD, 2014; Veldman *et al*., 2015). These findings could guide educational policy, particularly, integration of education and health sectors.

### Limitations

This study has some limitations. First, cohort participants came from two large urban areas and thus findings are more generalisable to urban areas, where more than 80% of the Brazilian population lives (OECD, 2014). Therefore, we only considered students who matched sample characteristics when applying our estimates to the Brazilian population (i.e. similar age group attending state-funded urban schools). As 72.8% of the Brazilian population attended state-funded urban schools in 2014, the number of negative educational outcomes avoided might be greater if we included individuals in private schools as well as those living in rural areas. Second, due to limitations of population data, the proportion of grade repetition, dropout and age-grade distortion were assumed equal between males and females, which might underestimate male and overestimate female rates. Third, given the mean sample age of 13.5 at follow-up, we are likely not capturing the peak rate of dropout (usually 15–17 years). This could explain why psychiatric conditions were more highly associated with repetition, which happens earlier and is a risk for later dropout. Fourth, we assumed psychiatric conditions have a causal impact on educational outcomes. We used a series of adjustments to improve causal inference including applying PSWs to minimise effects of potential confounders and adjusted for autoregressive effects. Nonetheless, unmeasured confounders, such as parental values and relationship of parents with children, family environment and social support could also explain these relationships. Fifth, bullying perpetration was assessed with one self-reported question, which may raise concern on its validity. The use of a prompt to explain and exemplify what is bullying may minimise inaccuracy. Moreover, single-item assessment of bullying has been demonstrated previously as being informative of health and service use outcomes over five decades (Takizawa *et al*., 2014; Evans-Lacko *et al*., 2017).

In conclusion, the current study estimated the impact of psychiatric conditions on educational outcomes, including examining different condition categories with several widely used educational indicators. The United Nation Sustainable Development Goals recognise education as ‘one of the most powerful vehicles for sustainable development’. Our findings provide support that treatment and prevention of psychiatric conditions could prevent around 4.9% of grade repetition and 7.7% of age-grade distortion in males and females, and 11.4% of bullying perpetration behaviour and low literacy performance in females with prior externalising-related conditions. Thus, policies which encourage early intervention and collaboration between education and health sectors to support the mental health of schoolchildren can have profound consequences for lifetime opportunities and socioeconomic well-being.

## Supplementary material

For supplementary material accompanying this paper visit http://dx.doi.org/10.1017/S2045796021000561. click here to view supplementary material

## Availability of data and materials

The data that support the findings of this study are available upon request in the *Open Science Framework* public repository (https://osf.io/ktz5h/) and syntax to process raw variables used in this study can also be found in *gitlab* (https://gitlab.com/bhrc/bhrc). Dr Hoffmann has full access to all the data used in the study, as well as analysis code (R and Stata), and takes responsibility for the integrity of the data and the accuracy of the data analysis.
