# Supplementary material for: The impact of child psychiatric conditions on future educational outcomes among a community cohort in Brazil
Source: Epidemiol Psychiatr Sci. 2021 Oct 28;30:e69. doi: 10.1017/S2045796021000561 (PMC8581950; doi:10.1017/S2045796021000561)
Supplement: Supplementary file 1 [file S2045796021000561sup001.docx]

**Online Supplementary Material**

**The impact of child psychiatric conditions on future educational outcomes among a community cohort in Brazil**

**Literacy – reading and writing ability**

The reading and writing test applied in this study (“Teste de Desempenho Escolar”) is described in the main paper and is validated to access these skills at the age-range of the present sample (Stein, 1998). We performed confirmatory factor analysis (CFA) using the *lavaan* package in R (Rosseel et al., 2018) to asses literacy at the trait level, according to what is applied in the Brazilian national high school exam (<http://download.inep.gov.br/educacao_basica/enem/nota_tecnica/2011/nota_tecnica_tri_enem_18012012.pdf>). All right (1) / wrong (0) answers of 12 reading items and 61 writing items on both time points were modelled to be loaded by one common literacy factor, clustered by subject. In this way, we could estimate the literacy performance in each time point and within-subject change across time. We used delta parameterization and weighted least square with diagonal weight matrix with standard errors and mean- and variance- adjusted chi-square test statistics (WLSMV) estimator and pairwise deletion of missing data. Model fit was tested by root mean square error of approximation (RMSEA), comparative fit index (CFI), and Tucker–Lewis index (TLI). RMSEA near or below 0.080 represent acceptable model fit, and values lower than 0.060 represent good-to-excellent model fit (Hu and Bentler, 1999). CFI and TLI values near or above 0.900 represent acceptable model fit, while values higher than 0.950 represent a good-to-excellent model fit (Hu and Bentler, 1999). Omega (ω) and Cronbach’s α coefficient were calculated to estimate factor reliability (Lucke, 2005; Raykov, 2001). CFA demonstrated excellent model fit and reliability indices (Hu and Bentler, 1999; Lucke, 2005) for this latent variable (RMSEA = 0.019, 90% CI 0.018-0.020; CFI 0.999; TLI 0.999; α = 0.995; ω = 0.984). Literacy factor scores were extracted to be used in main analysis.

**Brazilian educational data to estimate population attributable risk percentage**

For educational outcomes, we used data from the Brazilian National Education database (Instituto Nacional de Estudos e Pesquisas Educacionais (INEP) to estimate the number of Brazilians to which the PARPs would translate. In 2014, the final year of follow-up, 18,211,089 females and 18,661,803 males were enrolled in primary and secondary education (ages 6 to 17), which covers the educational period of cohort participants (http://portal.inep.gov.br/web/guest/sinopses-estatisticas-da-educacao-basica). To match the cohort profile, we restricted this number to students in urban and state-funded schools (73% of the Brazilian population in 2014). We used the 2014 Brazilian educational census (<http://portal.inep.gov.br/web/guest/indicadores-educacionais>) to estimate the proportion of students experiencing repetition (11.7%), dropout (5.4%) and age-grade distortion (28.7%). To be conservative, we applied event proportions for a one-year period only (i.e. we did not consider event rates for the cumulative period from 2010 to 2014). It is expected that age-grade distortions are higher than the sum of repetition and dropout in a given year, because this measure is cumulative in nature. In line with the age of cohort participants at follow-up (mean = 13.5 years; SD = 1.9) we applied event proportions for those in the final years of secondary education (11 to 14 years of age). For bullying perpetration, we applied gender stratified estimates of bullying perpetration for students in the 9^th^ grade (around age 14) from the 2015 National Survey of School Health (<https://www.ibge.gov.br/en/statistics/social/education/16837-national-survey-of-school-health-editions.html>): 24.2% of males and 15.6% of females reported bullying someone during the last month. Based on these estimates, we estimated the PARP for each adverse educational outcome.

**References**:

Hu, L. & Bentler, P. M. (1999) Cutoff criteria for fit indexes in covariance structure analysis: Conventional criteria versus new alternatives. *Structural Equation Modeling: A Multidisciplinary Journal*. [Online] 6 (1), 1–55.

Lucke, J. F. (2005) The alpha and the omega of congeneric test theory: An extension of reliability and internal consistency to heterogeneous tests. *Applied Psychological Measurement*. 29 (1), . [online]. Available from: https://works.bepress.com/joseph_lucke/19/ (Accessed 17 February 2017).

Raykov, T. (2001) Estimation of congeneric scale reliability using covariance structure analysis with nonlinear constraints. *British Journal of Mathematical and Statistical Psychology*. [Online] 54 (2), 315–323.

Rosseel, Y. et al. (2018) *lavaan: Latent Variable Analysis*. [online]. Available from: https://CRAN.R-project.org/package=lavaan (Accessed 15 December 2018).

Stein, L. M. (1998) *TDE Teste de Desempenho Escolar*. São Paulo: Casa do Psicólogo.

| **Table S1a - Absolute standardized difference of baseline variables before and after propensity score weighting for fear conditions in females** | | | | |
| --- | --- | --- | --- | --- |
|  | **No conditions before** | **No conditions after** | **Fear conditions** | **p-value** |
| Comorbid diagnostic group | 0.017 | 0.145 | 0.245 | 0.115 |
| Age (years) | 10.279 | 10.198 | 10.094 | 0.673 |
| São Paulo (Ref.Porto Alegre) | 0.497 | 0.323 | 0.276 | 0.358 |
| IQ (z-score) | -0.072 | -0.158 | -0.210 | 0.630 |
| SEG (ref. upper class) | 0.621 | 0.667 | 0.684 | 0.771 |
| Black/Mixid race/Indigenous (Ref.White/Asian) | 0.378 | 0.353 | 0.347 | 0.328 |
| Baseline grade repetition | 0.166 | 0.226 | 0.245 | 0.559 |
| Baseline school dropout | 0.020 | 0.024 | 0.031 | 0.475 |
| Baseline age-grade distortion | 0.142 | 0.203 | 0.235 | 0.577 |
| Baseline literacy ability | -0.414 | -0.531 | -0.582 | 0.633 |
| Baseline bully perpetration | 0.101 | 0.058 | 0.051 | 0.940 |
| Note: Propensity score weighting using average treatment effect on the treated method; IQ. intelligence quotient; SEG, socioeconomic group (middle-and-lower classes C/D/E compared against upper classes A/B). | | | | |

| **Table S1b - Absolute standardized difference of baseline variables before and after propensity score weighting for fear conditions in males** | | | | |
| --- | --- | --- | --- | --- |
|  | **No conditions before** | **No conditions after** | **Fear conditions** | **p-value** |
| Comorbid diagnostic group | 0.015 | 0.293 | 0.368 | 0.305 |
| Age (years) | 10.160 | 10.250 | 10.021 | 0.288 |
| São Paulo (Ref.Porto Alegre) | 0.534 | 0.391 | 0.342 | 0.389 |
| IQ (z-score) | 0.081 | 0.076 | -0.050 | 0.423 |
| SEG (ref. upper class) | 0.579 | 0.644 | 0.623 | 0.715 |
| Black/Mixid race/Indigenous (Ref.White/Asian) | 0.409 | 0.370 | 0.386 | 0.160 |
| Baseline grade repetition | 0.214 | 0.288 | 0.263 | 0.484 |
| Baseline school dropout | 0.018 | 0.060 | 0.070 | 0.559 |
| Baseline age-grade distortion | 0.187 | 0.265 | 0.281 | 0.780 |
| Baseline literacy ability | -0.569 | -0.502 | -0.502 | 0.999 |
| Baseline bully perpetration | 0.140 | 0.121 | 0.140 | 0.642 |
| Note: Propensity score weighting using average treatment effect on the treated method; IQ. intelligence quotient; SEG, socioeconomic group (middle-and-lower classes C/D/E compared against upper classes A/B). | | | | |

| **Table S2a - Absolute standardized difference of baseline variables before and after propensity score weighting for conditions group in females** | | | | |
| --- | --- | --- | --- | --- |
|  | **No conditions before** | **No conditions after** | **Distress conditions** | **p-value** |
| Comorbid diagnostic group | 0.011 | 0.227 | 0.441 | 0.011 |
| Age (years) | 10.220 | 10.932 | 10.932 | 1.000 |
| São Paulo (Ref.Porto Alegre) | 0.494 | 0.304 | 0.221 | 0.174 |
| IQ (z-score) | -0.085 | -0.142 | -0.061 | 0.592 |
| SEG (ref. upper class) | 0.625 | 0.621 | 0.647 | 0.717 |
| Black/Mixid race/Indigenous (Ref.White/Asian) | 0.377 | 0.384 | 0.338 | 0.519 |
| Baseline grade repetition | 0.165 | 0.279 | 0.294 | 0.643 |
| Baseline school dropout | 0.018 | 0.053 | 0.074 | 0.452 |
| Baseline age-grade distortion | 0.144 | 0.257 | 0.235 | 0.747 |
| Baseline literacy ability | -0.434 | -0.266 | -0.352 | 0.512 |
| Baseline bully perpetration | 0.095 | 0.116 | 0.132 | 0.904 |
| Note: Propensity score weighting using average treatment effect on the treated method; IQ. intelligence quotient; SEG, socioeconomic group (middle-and-lower classes C/D/E compared against upper classes A/B). | | | | |

| **Table S2b - Absolute standardized difference of baseline variables before and after propensity score weighting for distress conditions in males** | | | | |
| --- | --- | --- | --- | --- |
|  | **No conditions before** | **No conditions after** | **Distress conditions** | **p-value** |
| Comorbid diagnostic group | 0.018 | 0.425 | 0.552 | 0.23 |
| Age (years) | 10.14 | 10.064 | 10.312 | 0.468 |
| São Paulo (Ref.Porto Alegre) | 0.534 | 0.261 | 0.224 | 0.599 |
| IQ (z-score) | 0.068 | 0.072 | 0.105 | 0.843 |
| SEG (ref. upper class) | 0.574 | 0.689 | 0.746 | 0.46 |
| Black/Mixid race/Indigenous (Ref.White/Asian) | 0.408 | 0.465 | 0.388 | 0.409 |
| Baseline grade repetition | 0.211 | 0.367 | 0.358 | 0.765 |
| Baseline school dropout | 0.020 | 0.042 | 0.075 | 0.398 |
| Baseline age-grade distortion | 0.188 | 0.328 | 0.328 | 0.998 |
| Baseline literacy ability | -0.555 | -0.635 | -0.702 | 0.587 |
| Baseline bully perpetration | 0.136 | 0.128 | 0.209 | 0.144 |
| Note: Propensity score weighting using average treatment effect on the treated method; IQ. intelligence quotient; SEG, socioeconomic group (middle-and-lower classes C/D/E compared against upper classes A/B). | | | | |

| **Table S3a - Absolute standardized difference of baseline variables before and after propensity score weighting for externalizing conditions in females** | | | | |
| --- | --- | --- | --- | --- |
|  | **No conditions before** | **No conditions after** | **Externalizing conditions** | **p-value** |
| Comorbid diagnostic group | 0.008 | 0.148 | 0.262 | 0.106 |
| Age (years) | 10.263 | 10.236 | 10.264 | 0.895 |
| São Paulo (Ref.Porto Alegre) | 0.500 | 0.380 | 0.308 | 0.148 |
| IQ (z-score) | -0.058 | -0.292 | -0.274 | 0.885 |
| SEG (ref. upper class) | 0.630 | 0.629 | 0.600 | 0.594 |
| Black/Mixid race/Indigenous (Ref.White/Asian) | 0.377 | 0.339 | 0.362 | 0.333 |
| Baseline grade repetition | 0.153 | 0.237 | 0.323 | 0.067 |
| Baseline school dropout | 0.020 | 0.026 | 0.031 | 0.559 |
| Baseline age-grade distortion | 0.131 | 0.213 | 0.292 | 0.090 |
| Baseline literacy ability | -0.406 | -0.672 | -0.596 | 0.549 |
| Baseline bully perpetration | 0.089 | 0.109 | 0.154 | 0.325 |
| Note: Propensity score weighting using average treatment effect on the treated method; IQ. intelligence quotient; SEG, socioeconomic group (middle-and-lower classes C/D/E compared against upper classes A/B). | | | | |

| **Table S3b - Absolute standardized difference of baseline variables before and after propensity score weighting for externalizing conditions in males** | | | | |
| --- | --- | --- | --- | --- |
|  | **No conditions before** | **No conditions after** | **Externalizing conditions** | **p-value** |
| Comorbid diagnostic group | 0.011 | 0.18 | 0.211 | 0.658 |
| Age (years) | 10.163 | 10.119 | 10.073 | 0.799 |
| São Paulo (Ref.Porto Alegre) | 0.554 | 0.338 | 0.338 | 0.998 |
| IQ (z-score) | 0.109 | -0.218 | -0.124 | 0.540 |
| SEG (ref. upper class) | 0.568 | 0.591 | 0.658 | 0.199 |
| Black/Mixid race/Indigenous (Ref.White/Asian) | 0.408 | 0.422 | 0.404 | 0.384 |
| Baseline grade repetition | 0.194 | 0.320 | 0.342 | 0.486 |
| Baseline school dropout | 0.020 | 0.031 | 0.035 | 0.510 |
| Baseline age-grade distortion | 0.176 | 0.288 | 0.289 | 0.969 |
| Baseline literacy ability | -0.512 | -0.856 | -0.807 | 0.687 |
| Baseline bully perpetration | 0.123 | 0.216 | 0.224 | 0.929 |
| Note: Propensity score weighting using average treatment effect on the treated method; IQ. intelligence quotient; SEG, socioeconomic group (middle-and-lower classes C/D/E compared against upper classes A/B). | | | | |

| **Table S4 - Attrition-weighted unadjusted regression of diagnostic groups predicting educational outcomes by sex** | | | | | | | | | | | | |
| --- | --- | --- | --- | --- | --- | --- | --- | --- | --- | --- | --- | --- |
|  | **Grade repetition** | | **School dropout** | | **Age-grade distortion (years)** | | **Age-grade distortion (binary)** | | **Literacy** | | **Bully perpetrator** | |
|  | *OR* | *CI* | *OR* | *CI* | *IRR* | *CI* | *OR* | *CI* | *β* | *CI* | *OR* | *CI* |
| *Females* |  |  |  |  |  |  |  |  |  |  |  |  |
| Any Fear | 1.84** | [1.25,2.71] | 0.67 | [0.19,2.32] | 1.57** | [1.17,2.10] | 1.86** | [1.24,2.78] | -.24** | [-0.40,-0.08] | 1.29 | [0.55,3.01] |
| Any Distress | 2.03** | [1.23,3.35] | 1.85 | [0.64,5.37] | 1.83** | [1.29,2.59] | 1.80* | [1.03,3.15] | -0.11 | [-0.25,0.03] | 1.40 | [0.54,3.62] |
| Any Externalizing | 3.02*** | [1.82,5.03] | 1.45 | [0.62,3.38] | 2.14*** | [1.51,3.02] | 2.84*** | [1.68,4.79] | -0.32*** | [-0.48,-0.15] | 2.26** | [1.24,4.11] |
| *Males* |  |  |  |  |  |  |  |  |  |  |  |  |
| Any Fear | 1.39 | [0.92,2.12] | 1.91 | [0.87,4.22] | 1.27 | [0.91,1.78] | 1.69** | [1.15,2.49] | -0.09 | [-0.19,0.15] | 0.65 | [0.36,1.16] |
| Any Distress | 2.79** | [1.52,5.12] | 2.78 | [0.99,7.82] | 1.53* | [1.03,2.28] | 2.18** | [1.25,3.79] | -0.02 | [-0.20,0.15] | 1.21 | [0.55,2.68] |
| Any Externalizing | 2.56*** | [1.91,3.43] | 2.85*** | [1.69,4.82] | 2.15*** | [1.72,2.70] | 2.97*** | [2.15,4.10] | -0.22*** | [-0.81,-0.12] | 1.71* | [1.11,2.64] |
| Note: Models used only psychiatric condition group as predictors and were weighted using inverse probability weight for response in follow-up. OR, Odds ratio; CI, 95% confidence interval; IRR, incidence rate ratio; * p<0.05 ; ** p<0.01; *** p<0.001. | | | | | | | | | | | | |

| **Table 5a - Adjusted regression models of fear conditions predicting educational outcomes in females** | | | | | | | | | | | | |
| --- | --- | --- | --- | --- | --- | --- | --- | --- | --- | --- | --- | --- |
|  | **Grade repetition** | | **School dropout** | | **Age-grade distortion (years)** | | **Age-grade distortion (binary)** | | **Literacy** | | **Bully perpetrator** | |
| *Predictors with PSW* | *OR* | *CI* | *OR* | *CI* | *IRR* | *CI* | *OR* | *CI* | *β* | *CI* | *OR* | *CI* |
| Fear (PSW) | 1.42 | [0.82,2.46] | 0.37 | [0.09,1.43] | 1.22 | [0.80,1.87] | 1.33 | [0.72,2.48] | -0.06 | [-0.17,0.04] | 2.23 | [0.84,5.94] |
| Comorbid group (dummy) | 1.96 | [0.79,4.87] | 4.93* | [1.02,23.78] | 1.48 | [0.98,2.24] | 1.53 | [0.70,3.37] | -0.11 | [-0.27,0.06] |  |  |
| Age | 1.28** | [1.08,1.53] | 1.95*** | [1.51,2.51] | 1.11* | [1.00,1.23] | 1.13 | [0.98,1.31] | -0.05* | [-0.09,-0.01] | 1.11 | [0.88,1.40] |
| São Paulo (Ref.Porto Alegre) | 0.26** | [0.11,0.61] | 0.61 | [0.31,1.22] | 0.41** | [0.21,0.79] | 0.33* | [0.14,0.77] | 0.11 | [-0.04,0.27] | 0.39 | [0.11,1.42] |
| SEG (ref. upper class) | 0.99 | [0.45,2.16] | 0.68 | [0.23,2.05] | 0.96 | [0.52,1.77] | 0.82 | [0.35,1.92] | 0.06 | [-0.05,0.18] | 0.55 | [0.20,1.52] |
| Black/Mixid race/Indigenous (Ref.White/Asian) | 1.86 | [0.94,3.70] | 0.39 | [0.10,1.53] | 1.35 | [0.83,2.18] | 1.78 | [0.88,3.61] | -0.12 | [-0.27,0.02] | 0.88 | [0.27,2.90] |
| IQ (z-score) | 0.76 | [0.53,1.10] | 1.09 | [0.30,3.98] | 0.78 | [0.57,1.07] | 0.79 | [0.53,1.19] | 0.07* | [0.01,0.13] | 1.40 | [0.91,2.14] |
| Grade repetition at baseline | 1.80 | [0.89,3.62] |  |  |  |  |  |  |  |  |  |  |
| School dropout at baseline |  |  | 0.71 | [0.13,3.80] |  |  |  |  |  |  |  |  |
| Age-grade distortion (binary) at baseline |  |  |  |  | 1.25* | [1.04,1.51] |  |  |  |  |  |  |
| Age-grade distortion (years) at baseline |  |  |  |  |  |  | 1.64 | [0.75,3.59] |  |  |  |  |
| Literacy at baseline |  |  |  |  |  |  |  |  | 0.48*** | [0.38,0.59] |  |  |
| Bully perpetrator at baseline |  |  |  |  |  |  |  |  |  |  | 13.86*** | [5.32,36.12] |
| Observations | 806 | | 806 | | 808 | | 808 | | 646 | | 712 | |
| Note: Models were weighted using inverse probability weight for response in follow-up. PSW, propensity score weight using fear as treatment variable compared with no condition group. Comorbid group was not included in the model predicting bullying perpetration due to no cases of bullying perpetration in comorbid group. OR, Odds ratio; CI, 95% confidence interval; IRR, incidence rate ratio; SEG, socioeconomic group (upper classes A/B compared against middle-and-lower classes C/D/E); IQ, Intelligence quotient; * p<0.05 ; ** p<0.01; *** p<0.001. | | | | | | | | | | | | |

| **Table 5b - Adjusted regression models of fear conditions predicting educational outcomes in males** | | | | | | | | | | | | |
| --- | --- | --- | --- | --- | --- | --- | --- | --- | --- | --- | --- | --- |
|  | **Grade repetition** | | **School dropout** | | **Age-grade distortion (years)** | | **Age-grade distortion (binary)** | | **Literacy** | | **Bully perpetrator** | |
| *Predictors with PSW* | *OR* | *CI* | *OR* | *CI* | *IRR* | *CI* | *OR* | *CI* | *β* | *CI* | *OR* | *CI* |
| Fear (PSW) | 0.93 | [0.46,1.87] | 2.76* | [1.06,7.22] | 1.01 | [0.64,1.60] | 1.25 | [0.72,2.16] | -0.12 | [-0.24,0.01] | 0.57 | [0.22,1.52] |
| Comorbid group (dummy) | 1.91 | [0.91,3.99] | 1.30 | [0.37,4.55] | 1.07 | [0.61,1.90] | 1.41 | [0.66,3.00] | 0.00 | [-0.16,0.15] | 3.94** | [1.50,10.33] |
| Age | 1.18 | [0.95,1.47] | 1.98* | [1.11,3.53] | 1.08 | [0.94,1.24] | 1.02 | [0.84,1.24] | -0.03 | [-0.07,0.01] | 1.12 | [0.93,1.35] |
| São Paulo (Ref.Porto Alegre) | 0.29*** | [0.14,0.59] | 1.74 | [0.48,6.35] | 0.36*** | [0.21,0.62] | 0.33** | [0.17,0.63] | 0.04 | [-0.12,0.20] | 1.09 | [0.45,2.67] |
| SEG (ref. upper class) | 0.94 | [0.52,1.73] | 1.98 | [0.63,6.17] | 1.11 | [0.72,1.70] | 0.93 | [0.49,1.73] | -0.06 | [-0.17,0.05] | 0.50 | [0.20,1.24] |
| Black/Mixid race/Indigenous (Ref.White/Asian) | 1.23 | [0.59,2.59] | 0.50 | [0.17,1.44] | 1.01 | [0.60,1.71] | 1.23 | [0.59,2.60] | -0.06 | [-0.18,0.07] | 1.80 | [0.72,4.49] |
| IQ (z-score) | 0.77 | [0.55,1.09] | 0.86 | [0.57,1.30] | 0.88 | [0.68,1.15] | 0.82 | [0.60,1.13] | 0.04 | [-0.02,0.09] | 0.78 | [0.58,1.04] |
| Grade repetition at baseline | 2.27* | [1.07,4.78] |  |  |  |  |  |  |  |  |  |  |
| School dropout at baseline |  |  | 2.85 | [0.62,13.16] |  |  |  |  |  |  |  |  |
| Age-grade distortion (binary) at baseline |  |  |  |  | 1.12 | [0.88,1.41] |  |  |  |  |  |  |
| Age-grade distortion (years) at baseline |  |  |  |  |  |  | 1.38 | [0.68,2.79] |  |  |  |  |
| Literacy at baseline |  |  |  |  |  |  |  |  | 0.50*** | [0.41,0.59] |  |  |
| Bully perpetrator at baseline |  |  |  |  |  |  |  |  |  |  | 2.22 | [0.91,5.45] |
| Observations | 1010 | | 1010 | | 1014 | | 1014 | | 827 | | 937 | |
| Note: Models were weighted using inverse probability weight for response in follow-up. PSW, propensity score weight using fear as treatment variable compared with no condition group; OR, Odds ratio; CI, 95% confidence interval; IRR, incidence rate ratio; SEG, socioeconomic group (upper classes A/B compared against middle-and-lower classes C/D/E); IQ, Intelligence quotient; * p<0.05 ; ** p<0.01; *** p<0.001. | | | | | | | | | | | | |

| **Table 6a - Adjusted regression models of distress conditions predicting educational outcomes in females** | | | | | | | | | | | | | |
| --- | --- | --- | --- | --- | --- | --- | --- | --- | --- | --- | --- | --- | --- |
|  | **Grade repetition** | | **School dropout** | | **Age-grade distortion (years)** | | **Age-grade distortion (binary)** | | **Literacy** | | **Bully perpetrator** | | |
| *Predictors with PSW* | *OR* | *CI* | *OR* | *CI* | *IRR* | *CI* | *OR* | *CI* | *β* | *CI* | *OR* | *CI* |  |
| Distress (PSW) | 0.96 | [0.51,1.81] | 1.61 | [0.48,5.35] | 0.89 | [0.58,1.36] | 0.85 | [0.43,1.68] | -0.04 | [-0.17,0.08] | 2.08 | [0.79,5.47] |  |
| Comorbid group (dummy) | 2.57 | [0.79,8.39] | 1.65 | [0.30,9.00] | 1.58* | [1.02,2.45] | 1.81 | [0.73,4.47] | -0.15 | [-0.33,0.03] |  |  |  |
| Age | 1.24* | [1.03,1.49] | 1.45 | [0.99,2.14] | 1.08 | [0.94,1.23] | 1.00 | [0.85,1.18] | -0.05 | [-0.10,0.01] | 1.33 | [0.89,2.00] |  |
| São Paulo (Ref.Porto Alegre) | 0.52 | [0.18,1.49] | 1.35 | [0.33,5.49] | 0.71 | [0.34,1.49] | 0.89 | [0.31,2.54] | .096 | [-0.08 ,0.27] | 1.18 | [0.36,3.92] |  |
| SEG (ref. upper class) | 0.64 | [0.30,1.37] | 0.49 | [0.15,1.58] | 1.04 | [0.60,1.80] | 0.68 | [0.27,1.72] | 0.14 | [-0.00,0.27] | 1.78 | [0.43,7.36] |  |
| Black/Mixid race/Indigenous (Ref.White/Asian) | 0.77 | [0.35,1.67] | 1.18 | [0.27,5.22] | 0.98 | [0.58,1.65] | 0.91 | [0.38,2.19] | -0.05 | [-0.21,0.10] | 1.84 | [0.38,9.02] |  |
| IQ (z-score) | 0.69* | [0.48,0.98] | 1.38 | [0.52,3.72] | 0.74 | [0.51,1.07] | 0.93 | [0.60,1.45] | 0.03 | [-0.04,0.12] | 0.97 | [0.33,2.82] |  |
| Grade repetition at baseline | 3.24** | [1.43,7.34] |  |  |  |  |  |  |  |  |  |  |  |
| School dropout at baseline |  |  | 0.93 | [0.10,8.90] |  |  |  |  |  |  |  |  |  |
| Age-grade distortion (binary) at baseline |  |  |  |  | 1.19 | [0.94,1.50] |  |  |  |  |  |  |  |
| Age-grade distortion (years) at baseline |  |  |  |  |  |  | 2.06 | [0.72,5.90] |  |  |  |  |  |
| Literacy at baseline |  |  |  |  |  |  |  |  | 0.42*** | [0.28 ,0.55] |  |  |  |
| Bully perpetrator at baseline |  |  |  |  |  |  |  |  |  |  | 1.18 | [0.24,5.70] |  |
| Observations | 806 | | 806 | | 808 | | 808 | | 646 | | 712 | | |
| Note: Models were weighted using inverse probability weight for response in follow-up. PSW, propensity score weight using distress as treatment variable compared with no condition group. Comorbid group was not included in the model predicting bullying perpetration due to no cases of bullying perpetration in comorbid group. OR, Odds ratio; CI, 95% confidence interval; IRR, incidence rate ratio; SEG, socioeconomic group (upper classes A/B compared against middle-and-lower classes C/D/E); IQ, Intelligence quotient; * p<0.05 ; ** p<0.01; *** p<0.001. | | | | | | | | | | | | | |

| **Table 6b - Adjusted regression models of distress conditions predicting educational outcomes in males** | | | | | | | | | | | | |
| --- | --- | --- | --- | --- | --- | --- | --- | --- | --- | --- | --- | --- |
|  | **Grade repetition** | | **School dropout** | | **Age-grade distortion (years)** | | **Age-grade distortion (binary)** | | **Literacy** | | **Bully perpetrator** | |
| *Predictors with PSW* | *OR* | *CI* | *OR* | *CI* | *IRR* | *CI* | *OR* | *CI* | *β* | *CI* | *OR* | *CI* |
| Distress (PSW) | 2.76** | [1.32,5.78] | 3.16 | [0.79,12.64] | 1.25 | [0.77,2.05] | 1.85 | [0.92,3.71] | 0.16 | [-0.01,0.32] | 2.02 | [0.77,5.33] |
| Comorbid group (dummy) | 1.01 | [0.42,2.45] | 0.77 | [0.10,5.80] | 1.04 | [0.59,1.83] | 0.98 | [0.36,2.73] | -0.26*** | [-0.39,-0.13] | 1.48 | [0.61,3.63] |
| Age | 1.30** | [1.08,1.58] | 1.59** | [1.21,2.10] | 1.01 | [0.89,1.14] | 0.86 | [0.72,1.04] | -0.03 | [-0.09,0.02] | 0.88 | [0.70,1.10] |
| São Paulo (Ref.Porto Alegre) | 0.23* | [0.07,0.74] | 0.91 | [0.11,7.40] | 0.30*** | [0.17,0.56] | 0.23** | [0.09,0.57] | 0.04 | [-0.19,0.27] | 2.16 | [0.95,4.93] |
| SEG (ref. upper class) | 0.66 | [0.29,1.53] | 1.09 | [0.25,4.82] | 0.82 | [0.48,1.42] | 0.73 | [0.27,1.97] | 0.10 | [-0.06,0.26] | 0.31* | [0.10,0.99] |
| Black/Mixid race/Indigenous (Ref.White/Asian) | 1.27 | [0.54,3.01] | 0.14* | [0.02,0.81] | 0.82 | [0.44,1.50] | 0.91 | [0.35,2.40] | 0.07 | [-0.10,0.24] | 1.28 | [0.49,3.35] |
| IQ (z-score) | 0.59* | [0.38,0.91] | 0.28*** | [0.15,0.52] | 0.63** | [0.47,0.86] | 0.51* | [0.30,0.87] | 0.03 | [-0.01,0.08] | 0.70* | [0.49,0.99] |
| Grade repetition at baseline | 0.63 | [0.16,2.51] |  |  |  |  |  |  |  |  |  |  |
| School dropout at baseline |  |  | 21.84** | [2.73,174.95] |  |  |  |  |  |  |  |  |
| Age-grade distortion (binary) at baseline |  |  |  |  | 0.86 | [0.63,1.18] |  |  |  |  |  |  |
| Age-grade distortion (years) at baseline |  |  |  |  |  |  | 0.69 | [0.30,1.63] |  |  |  |  |
| Literacy at baseline |  |  |  |  |  |  |  |  | 0.57*** | [0.46,0.69] |  |  |
| Bully perpetrator at baseline |  |  |  |  |  |  |  |  |  |  | 1.35 | [0.37,4.98] |
| Observations | 1010 | | 1010 | | 1014 | | 1014 | | 827 | | 937 | |
| Note: Models were weighted using inverse probability weight for response in follow-up. PSW, propensity score weight using distress as treatment variable compared with no condition group; OR, Odds ratio; CI, 95% confidence interval; IRR, incidence rate ratio; SEG, socioeconomic group (upper classes A/B compared against middle-and-lower classes C/D/E); IQ, Intelligence quotient; * p<0.05 ; ** p<0.01; *** p<0.001. | | | | | | | | | | | | |

| **Table 7a - Multivariable regression models of externalizing disorders predicting educational outcomes in females** | | | | | | | | | | | | |
| --- | --- | --- | --- | --- | --- | --- | --- | --- | --- | --- | --- | --- |
|  | **Grade repetition** | | **School dropout** | | **Age-grade distortion (years)** | | **Age-grade distortion (binary)** | | **Literacy** | | **Bully perpetrator** | |
| *Predictors with PSW* | *OR* | *CI* | *OR* | *CI* | *IRR* | *CI* | *OR* | *CI* | *β* | *CI* | *OR* | *CI* |
| Externalizing (PSW) | 2.03* | [1.15,3.58] | 0.59 | [0.16,2.18] | 2.02** | [1.28,3.17] | 2.88*** | [1.61,5.14] | -0.23*** | [-0.34,-0.12] | 3.12** | [1.50,6.51] |
| Comorbid group (dummy) | 1.89 | [0.65,5.46] | 3.81 | [0.78,18.56] | 1.05 | [0.64,1.72] | 0.95 | [0.45,2.00] | -0.01 | [-0.15,0.14] |  |  |
| Age | 1.34** | [1.11,1.61] | 2.15*** | [1.51,3.05] | 1.05 | [0.95,1.16] | 1.06 | [0.91,1.23] | -0.05* | [-0.09,-0.01] | 1.12 | [0.93,1.35] |
| São Paulo (Ref.Porto Alegre) | 0.41* | [0.19,0.89] | 2.00 | [0.64,6.27] | 0.51* | [0.30,0.86] | 0.56 | [0.26,1.21] | -0.03 | [-0.14,0.09] | 0.78 | [0.27,2.24] |
| SEG (ref. upper class) | 1.72 | [0.89,3.30] | 0.62 | [0.19,1.98] | 1.32 | [0.81,2.15] | 1.28 | [0.60,2.73] | 0.12* | [0.00,0.24] | 1.74 | [0.67,4.53] |
| Black/Mixid race/Indigenous (Ref.White/Asian) | 1.27 | [0.65,2.50] | 0.25 | [0.06,1.06] | 0.92 | [0.60,1.41] | 1.07 | [0.61,1.89] | -0.07 | [-0.17,0.04] | 0.74 | [0.24,2.22] |
| IQ (z-score) | 0.76 | [0.55,1.06] | 0.71 | [0.33,1.52] | 0.85 | [0.66,1.09] | 0.92 | [0.64,1.34] | 0.07 | [-0.01,0.15] | 1.05 | [0.68,1.63] |
| Grade repetition at baseline | 1.96* | [1.14,3.38] |  |  |  |  |  |  |  |  |  |  |
| School dropout at baseline |  |  | 1.10 | [0.12,10.47] |  |  |  |  |  |  |  |  |
| Age-grade distortion (binary) at baseline |  |  |  |  | 1.16 | [0.96,1.42] |  |  |  |  |  |  |
| Age-grade distortion (years) at baseline |  |  |  |  |  |  | 1.51 | [0.78,2.91] |  |  |  |  |
| Literacy at baseline |  |  |  |  |  |  |  |  | 0.48*** | [0.39,0.57] |  |  |
| Bully perpetrator at baseline |  |  |  |  |  |  |  |  |  |  | 1.09 | [0.41,2.88] |
| Observations | 806 | | 806 | | 808 | | 808 | | 646 | | 712 | |
| Note: Models were weighted using inverse probability weight for response in follow-up. PSW, propensity score weight using externalizing as treatment variable compared with no condition group. Comorbid group was not included in the model predicting bullying perpetration due to no cases of bullying perpetration in comorbid group. OR, Odds ratio; CI, 95% confidence interval; IRR, incidence rate ratio; SEG, socioeconomic group (upper classes A/B compared against middle-and-lower classes C/D/E); IQ, Intelligence quotient; * p<0.05 ; ** p<0.01; *** p<0.001. | | | | | | | | | | | | |

| **Table 7b - Adjusted regression models of externalizing conditions predicting educational outcomes in males** | | | | | | | | | | | | |
| --- | --- | --- | --- | --- | --- | --- | --- | --- | --- | --- | --- | --- |
|  | **Grade repetition** | | **School dropout** | | **Age-grade distortion (years)** | | **Age-grade distortion (binary)** | | **Literacy** | | **Bully perpetrator** | |
| *Predictors with PSW* | *OR* | *CI* | *OR* | *CI* | *IRR* | *CI* | *OR* | *CI* | *β* | *CI* | *OR* | *CI* |
| Externalizing (PSW) | 1.66* | [1.05,2.64] | 1.24 | [0.51,3.01] | 1.39* | [1.06,1.83] | 1.66* | [1.05,2.62] | 0.00 | [-0.09,0.09] | 1.36 | [0.74,2.47] |
| Comorbid group (dummy) | 1.39 | [0.79,2.46] | 2.16 | [0.49,9.59] | 0.77 | [0.48,1.24] | 1.10 | [0.61,1.95] | -0.03 | [-0.16,0.09] | 1.12 | [0.57,2.23] |
| Age | 1.26*** | [1.12,1.42] | 1.82*** | [1.41,2.35] | 1.06 | [0.99,1.14] | 0.99 | [0.86,1.12] | -0.05* | [-0.08,-0.01] | 1.07 | [0.93,1.24] |
| São Paulo (Ref.Porto Alegre) | 0.22*** | [0.13,0.37] | 1.18 | [0.47,2.98] | 0.35*** | [0.25,0.49] | 0.32*** | [0.20,0.52] | 0.00 | [-0.12,0.13] | 1.26 | [0.69,2.30] |
| SEG (ref. upper class) | 0.83 | [0.56,1.23] | 2.64 | [0.98,7.07] | 1.10 | [0.90,1.34] | 0.93 | [0.64,1.35] | -0.07 | [-0.15,0.01] | 1.25 | [0.72,2.19] |
| Black/Mixid race/Indigenous (Ref.White/Asian) | 1.42 | [0.88,2.30] | 0.67 | [0.25,1.82] | 1.03 | [0.77,1.36] | 1.22 | [0.70,2.13] | -0.06 | [-0.15,0.02] | 0.97 | [0.51,1.85] |
| IQ (z-score) | 0.66** | [0.51,0.86] | 0.70 | [0.44,1.13] | 0.82 | [0.66,1.01] | 0.73* | [0.56,0.95] | .04 | [-0.01,0.08] | 0.99 | [0.82,1.20] |
| Grade repetition at baseline | 1.24 | [0.72,2.15] |  |  |  |  |  |  |  |  |  |  |
| School dropout at baseline |  |  | 1.10 | [0.23,5.29] |  |  |  |  |  |  |  |  |
| Age-grade distortion (binary) at baseline |  |  |  |  | 1.05 | [0.92,1.20] |  |  |  |  |  |  |
| Age-grade distortion (years) at baseline |  |  |  |  |  |  | 1.35 | [0.77,2.37] |  |  |  |  |
| Literacy at baseline |  |  |  |  |  |  |  |  | 0.50*** | [0.43,0.56] |  |  |
| Bully perpetrator at baseline |  |  |  |  |  |  |  |  |  |  | 1.85* | [1.04,3.28] |
| Observations | 1010 | | 1010 | | 1014 | | 1014 | | 827 | | 937 | |
| Note: Models were weighted using inverse probability weight for response in follow-up. PSW, propensity score weight using externalizing as treatment variable compared with no condition group; OR, Odds ratio; CI, 95% confidence interval; IRR, incidence rate ratio; SEG, socioeconomic group (upper classes A/B compared against middle-and-lower classes C/D/E); IQ, Intelligence quotient; * p<0.05 ; ** p<0.01; *** p<0.001. | | | | | | | | | | | | |
